# Non-communicable disease care for persons living with HIV in Peru: A national physician cross-sectional study

**DOI:** 10.1371/journal.pgph.0004846

**Published:** 2025-08-04

**Authors:** Rebecca Slotkin, Daniel Granda, Diego Cabrera, Carlos Manuel Benites, Patricia J. Garcia, Evelyn Hsieh

**Affiliations:** 1 Johns Hopkins University, Department of Medicine, Division of Rheumatology, Baltimore, Maryland, United States of America; 2 Yale School of Public Health, New Haven, Connecticut, United States of America; 3 Yale School of Medicine, Department of Medicine, Section of Rheumatology, New Haven, Connecticut, United States of America; 4 Facultad de Medicina Alberto Hurtado de la Universidad Peruana Cayetano Heredia, Lima, Peru; 5 Yale New Haven Health System, Department of Internal Medicine, Waterbury Hospital, Waterbury Connecticut, United States of America; 6 Ministerio de Salud, Lima, Peru; 7 Epidemiology, STD, and HIV Unit, School of Public Health, Universidad Peruana Cayetano Heredia, Lima, Peru; 8 VA Connecticut Healthcare System, West Haven, Connecticut, United States of America; Tribhuvan University Institute of Medicine, NEPAL

## Abstract

Non-communicable diseases (NCDs) are a significant cause of morbidity and mortality for the aging HIV population worldwide. In Peru, no data exists on how providers address NCDs for persons living with HIV (PLWH). This study examines HIV physician confidence and current management practices for NCDs for PLWH in Peru. We recruited public-sector HIV physicians via Peru’s National HIV, STI and Hepatitis Program’s (NHSTIHP) physician registry and by program coordinator referral. Participants completed a telephone survey encompassing seven NCDs [hyperlipidemia, hypertension, diabetes, osteoporosis, sarcopenia, non-AIDS defining cancers, neurocognitive impairment (NCI)] and three modifiable risk factors (obesity, tobacco, and alcohol use). Survey domains included: (1) *provider and practice characteristics* (2) *NCDs encountered,* (3) *provider confidence* in prevention, diagnosis, and treatment (based upon a four-point Likert scale), (4) *screening frequency and management approaches* (free response). We obtained contact information from 167 physicians working with the NHSTIHP, and 78 (47%) volunteered to participate (mean age 45.8 ± 9.3 years; 26% women; 78% infectious disease trained) across 23 of the 25 regions of Peru. The majority (>50%) of physicians reported at least one patient with: hyperlipidemia, hypertension, diabetes, NCI, cervical cancer, obesity, tobacco, and/or alcohol use. Physicians felt most confident independently managing metabolic disorders (hyperlipidemia, diabetes, hypertension, obesity), and least confident with NCI and sarcopenia. Most physicians (>50%) would manage the NCDs, although management approaches differed. NCD screening that was part of the NHSTIHP National HIV care guidelines was more consistently performed than screening beyond the scope of the existing guidelines. Peruvian HIV physicians encounter NCDs in their patient population and manage these conditions and risk factors despite variable confidence and/or knowledge of best practices. This study highlights opportunities for expanding physician education, addressing systems-level barriers to NCD care, and the need for locally relevant, epidemiologically-based, HIV-specific NCD care guidelines.

## Introduction

As persons living with HIV (PLWH) on antiretroviral therapy (ART) age, they face an added burden of non-communicable diseases (NCD) from HIV-related inflammation, ART toxicity, and the aging process itself [1–4]. As a result, the morbidity and mortality trends among PLWH in the Latin American region have gradually shifted toward NCDs and away from AIDS-defining illness in recent years [2,5,6]. Although 70% of deaths annually are attributed to NCDs in the Peruvian general population, similar data for PLWH in Peru is lacking [7]. Studies have demonstrated that PLWH have a high prevalence of NCDs such as diabetes, cardiovascular disease, low bone density, renal disease and non-AIDS defining cancers (i.e., breast, colon, anal and cervical) [3,4,8–11]. Many of these diseases and risk factors for these diseases are manageable and modifiable in a primary care setting. Without a parallel focus on the successful management of NCDs, PLWH will not be able to experience the full potential of the health gains from advancements in ART.

In Peru, there are an estimated 110,000 PLWH [12]. According to 2020 Ministry of Health data, approximately 77% of PLWH received their HIV care from providers employed by the Ministry of Health’s National HIV/STI and Hepatitis Program (NHSTIHP) and of those receiving care, 64% were over the age of 30 [13]. Physicians see their patients every three months to distribute ART and are the primary point of medical care for PLWH. Although the Peruvian National HIV guidelines for routine HIV care include basic screening recommendations for diabetes, hyperlipidemia, breast and cervical cancer, there are no national practice guidelines to address management of NCDs for PLWH in Peru [14].

Studies have demonstrated the clinical effectiveness and cost-effectiveness of integrating NCD care for PLWH [15,16]. In a cross-sectional study from Brazil examining predictors of success with integrating HIV education and services into primary care, the authors found that provider knowledge, skills and confidence predicted successful integration [17]. Little is known about provider confidence and the current management practices for NCDs in PLWH in Peru. This study partnered with the NHSTIHP to investigate the HIV physician experience and current management of NCDs in PLWH in Peru.

## Methods

### Study design and ethics

This was a cross-sectional, telephone survey-based study conducted in Peru. The study was approved by the Ethics Review Committee of the Universidad Peruana Cayetano Heredia (#206475) on September 8^th^ 2021 and issued an exemption from Yale University’s IRB (#2000031131) on October 11^th^ 2021. Verbal consent was obtained over the phone prior to participation in any study activities as approved by the Cayetano IRB. A copy of the consent and study description documents in Spanish were sent to participants. Participants were contacted and recruited starting November 25^th^ 2021 through June 30^th^ 2022.

### Study setting

The healthcare system in Peru is segmented by four main funding/insurance entities. The Seguro Integral de Salud (SIS) is a Ministry of Health program, which is funded by general taxes, provides free/lower priced healthcare for those in poverty, and covers approximately 65% of the population [18]. Access to health services depends on an individual’s insurance and location within the country. Although there is a network of primary health care centers, half of the centers do not have a doctor, and most do not have adequate infrastructure or internet [18]. In 2019, only 29% of Peruvians would go to a primary health center first for a health need [18], the majority instead seeking care directly from hospitals or pharmacies [19].

The Ministry of Health’s NHSTIHP program has a network of providers and coordinators across the country, who provide health services for the majority of PLWH. Regional program coordinators have a multifaceted administrative role, which involves performing administrative tasks such as budget allocation and supply/medication requests, supervising region-specific HIV programming, running community and provider education campaigns, and overseeing service delivery. They are also in direct contact with the HIV physicians in their region and/or with local level coordinators.

### Study population and recruitment

All HIV physicians working for the Ministry of Health’s NHSTIHP who predominantly saw PLWH covered by SIS and had seen at least one PLWH within the last year were eligible for participation. Providers were identified via the Program’s physician registry and through referrals from regional NHSTIHP coordinators. An email from the NHSTIHP was sent to the regional program coordinators notifying them of the study. These coordinators provided additional physician contact information beyond the Ministry’s physician registry. Physicians and coordinators were contacted by a trained bilingual Peruvian research assistant in Spanish via WhatsApp and email, as approved by the IRB, and sent an informational message about the study purpose and procedures, including a copy of the consent form. If they could confirm they met the eligibility criteria and were willing to participate, they were contacted for the survey.

### Data collection and measures

All participants completed a telephone survey conducted and recorded in Spanish administered by the same trained Peruvian research assistant, who documented the responses electronically in the study database using *Qualtrics Research Suite* through Yale University’s enterprise license*.* The data was stored in *Qualtrics* and Yale’s secure *Box*. The survey tool included 35 items divided into five domains: (1) *provider and practice characteristics* (2) *provider perception of NCDs encountered in practice,* (3) *provider confidence in* prevention, diagnosis, and treatment of NCDs (based upon a four-point Likert scale), (4) *NCD screening frequency,* and (5) *NCD management approaches* (free response).

Seven NCDs [hyperlipidemia (HLD), hypertension (HTN), diabetes (DM), osteoporosis, sarcopenia, non-AIDS defining cancer (breast, cervical, anal, prostate, colon), neurocognitive impairment (NCI)] and three modifiable risk factors (obesity, tobacco, and alcohol use) were addressed in the study survey. The NCDs chosen for this study fell into the one of the following categories: 1) modifiable diseases with high morbidity and/or mortality with known or suspected increased prevalence in HIV and 2) modifiable risk factors that contribute to the development of other non-communicable diseases (S1 Table, S1 Data).

Provider and practice questions collected the following data: physician age, sex, primary practice location (coast, highlands, jungle), number of years in practice, type of training (general medicine, internal medicine, infectious disease), number of HIV patients seen in the last month, and perceived percentage of HIV patients >40 years of age. Primary clinical site care level (I, II, III) was determined through publicly available websites and ministry data. NCDs encountered in a provider’s patient population was assessed by asking if “any of their HIV patients have any of the following conditions” for each of the 7 NCDs and 3 risk factors included in the study. Provider confidence with prevention, diagnosis, and treatment of each NCD and risk factor was assessed on a four-point Likert scale [1-no confidence, 2- little confidence, 3- confident, 4- very confident] and scored separately (S1 Data). Survey questions are available in the supplemental documents (S1 Text, S2 Text).

The questions regarding screening frequency and management approaches were created based on guidelines compiled from the World Health Organization (WHO), United States Preventative Services Task Force (USPSTF), Infectious Diseases Society of America (IDSA) Primary Care Guidelines, and European Consensus Guidelines [17,18,20–22]. Where guidelines differed, screening and management approaches were discussed with Peruvian physicians. The questions focused on primary prevention when possible. NCD screening questions were phrased as multiple-choice questions for HTN, HLD, DM, obesity, osteoporosis, sarcopenia and NCI. Cancer screening practices were assessed via free response questions divided by patient sex at birth. Questions regarding NCD management were asked in an open-ended format and the research assistant recorded providers’ responses using pre-designated categories generated during pilot testing of the survey. Categories were verified by the research assistant to minimize interpretive errors. The pre-designated categories were not visible or described to respondents at the time of the telephone call to avoid biasing their responses. Free responses that did not fit into any of the predesignated categories were documented by the research assistant in the “other” section. The “other” section was translated to English by the original bilingual Peruvian research assistant who recorded them. These responses were grouped into categories, tallied by a separate researcher and double checked for accuracy by the original research assistant who conducted the survey.

### Statistical analysis

All analyses were performed using *RStudio* 2022.07.1. Descriptive statistics were used to report physician demographics, clinical site characteristics and to categorize free responses for physician confidence, screening frequency, and management practices. Data with normal distribution was reported with mean + /- SD, non-normally distributed data with median and interquartile range (IQR). Physician confidence was grouped for analysis into *low confidence* (Likert scale 1 and 2) and *high confidence* (Likert scale 3 and 4). *Direct management* by the primary HIV provider was defined as any of the following activities: counseling, ordering labs or imaging, reviewing, and changing medication regimens as appropriate, pharmacologic interventions, and referral to non-physician ancillary services (ex: nutrition and physical therapy). Primary HIV providers who both performed direct management activities and referred patients to another physician specialist for support were counted as providing initial management. Subcategories of initial management included those who managed without placing referrals and those who managed while placing referrals. Physicians who did not take any management actions themselves, but did place referrals to other providers for care were separated into a “specialist referral only” category.

## Results

### Provider characteristics

**Fig 1:**
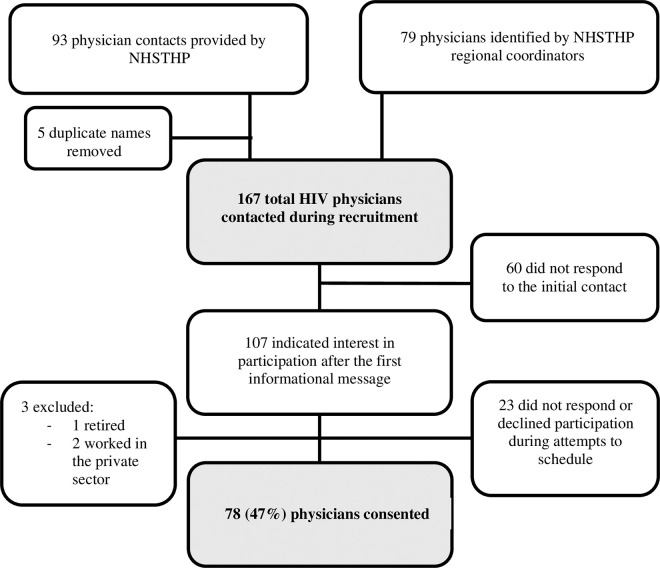
Participant recruitment flow diagram.

The NHSTIHP provided a list of 93 physicians providing HIV care in Peru with five duplicates, which were removed. Of twenty-five total regional HIV program coordinators in Peru, 21 provided support to the project by providing contact information for an additional 79 physicians working with the NHSTIHP. Therefore, a total of 167 physicians were contacted during recruitment, and 107 responded with interest to an initial informational message about the study. Twenty-three physicians subsequently declined to participate and three were excluded (one had retired and two no longer worked with the Ministry). Seventy-eight volunteered to participate in the survey providing a 47% response rate (Fig 1).

The mean age of study participants was 45.8 ± 9.3, median age 46 years. The majority were infectious disease trained and from the coastal region. The physician participants were from both urban and rural practices across all regions of Peru, representing a range of health center levels from primary clinics to complex-care referral hospitals. Physicians reported seeing a median of 62.5 (IQR 16–150) HIV patients per month, had been in practice a median of 17.5 years (IQR 11–25) (Table 1).

**Table 1:** Participants Characteristics and Perceptions of Non-communicable Diseases (N = 78).

Variable	Physician Response
**Age** *mean ± SD [median]*	45.8 ± 9.3 [46]
**Women** *n (%)*	20 (26%)
**Number of Years in Practice** *median (IQR)*	17.5 (11-25)
**Region** *n (%)*	
Coast	48 (61%)
Highlands	14 (18%)
Jungle	16 (21%)
**Regions Represented** *n (%)*	23 (92%)
**Professional Training** *n (%)*	
Infectious Disease	61 (78%)
** **Generalist or Internist	17 (22%)
**Practice Site Level *n (%)***	
** **Primary Health Center	30 (40%)
** **Small Hospital	31 (42%)
** **Tertiary Care Hospital	13 (18%)
** Self-Reported Number of HIV Patients Seen per Month** *median (IQR)*	62.5 (16-150)
** Estimated Percent of Patients >40 Years Old** *median (IQR)*	40% (30%-50%)

### Provider confidence and NCDs encountered in practice

The self-reported diseases/risk factors that most (>50%) physicians encountered in their patients with HIV were: hyperlipidemia, alcohol use, diabetes, obesity, hypertension, neurocognitive impairment, tobacco use, and cervical cancer. Physicians least frequently reported having any HIV patients with sarcopenia, anal, colon, and breast cancer (S2 Table).

**Fig 2:**
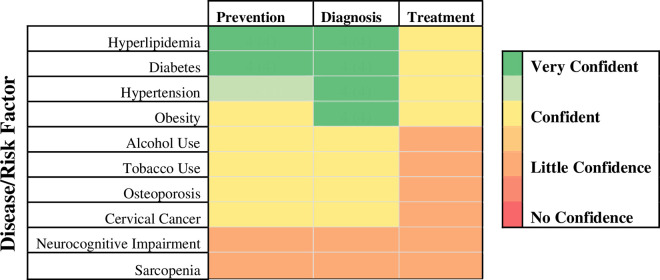
Physician Self-reported Median Confidence Score Heatmap. Physicians rated their confidence on a 4-point Likert scale (no confidence, little confidence, confident, very confident) in the domains of prevention, diagnosis, and treatment for the above non-communicable diseases/risk factors with respect to caring for their HIV patients.

When asked to rate their confidence with prevention, diagnosis, and treatment of the selected NCDs, physicians were most confident managing metabolic diseases (HLD, DM, HTN, obesity). Physicians were least confident with osteoporosis, cervical cancer, NCI, and sarcopenia. In general, physicians were more confident with prevention and diagnosis rather than treatment of the diseases and risk factors discussed (Fig 2).

### Screening practices and management approaches

**Fig 3:**
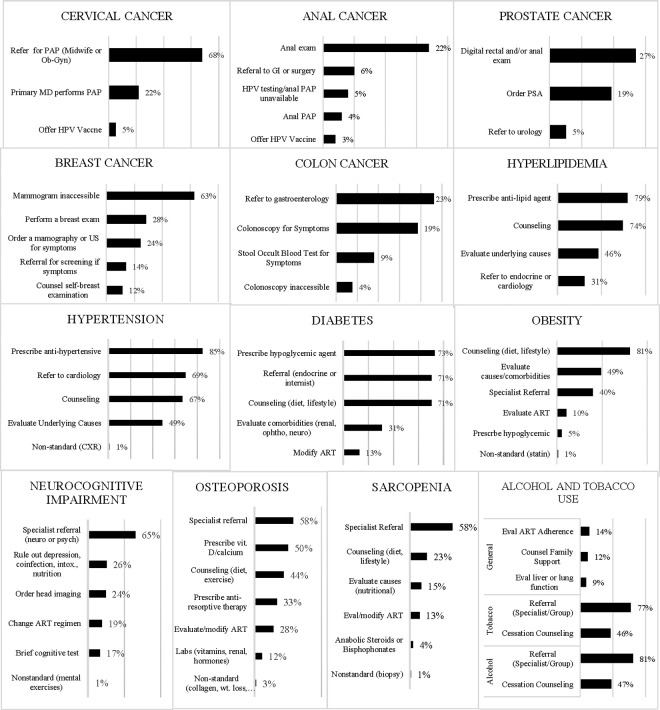
Physicians were asked what they would do if they had a patient with the above diseases or risk factors. The free responses were grouped into non-exclusive categories to examine practice patterns. The x-axis depicts the percent of physicians who responded with that type of management. The y-axis depicts types of management practices. Physicians shown here as asking for specialist referral also did at least one other form of NCD management listed such as counseling, adjusting ART, or starting initial therapy.

The self-reported screening and management practices were characterized in Table 2. Physicians were least likely to address cancer screening with their HIV patients: 96% reported that they do not address anogenital cancer screening, 73% do not address prostate cancer screening, 58% do not address colon cancer screening, 45% do not address breast cancer screening, and 22% do not address cervical cancer screening (Table 2A). For non-cancer conditions, physicians were least likely to address sarcopenia (15%), followed by obesity (8%), alcohol (8%), and tobacco use (8%). Physicians were most likely to manage hyperlipidemia by themselves (71%) without needing any additional physician referral, followed by diabetes (36%), hypertension (32%), and obesity (32%) (Table 2B). Although most physicians reported either direct management and/or referral for the selected NCDs and disease risk factors in PLWH, specific management practices differed widely between physicians. Many physicians reported more than one management strategy. Specific management practices by disease/risk factor were grouped and are illustrated with non-exclusive categorization in Fig 3.

**Table 2:** Self-Reported HIV Physician NCD Screening and Management for PLWH (N = 78).

A. Self-Reported Cancer Screening Approaches among HIV Providers									
Cancer Screening				Primary MD Screens		Specialist (Midwife or MD) Referral for Screening		No Management	
Anogenital Cancer Screening (anal PAP)				2 (3%)		1 (1%)		75 (96%)	
Cervical Cancer Screening (cervical PAP)				8 (10%)		53 (68%)		17 (22%)	
Prostate Cancer Screening (PSA, DRE)				17 (22%)		4 (5%)		57 (73%)	
**Symptom** Driven Colon cancer Screening (Fecal Occult Blood, Colonoscopy)				16 (21%)		17 (22%)		45 (58%)	
**Symptom** Driven Breast Cancer Screening (Breast Exam, Breast U/S, Mammography)				32 (41%)		11 (14%)		35 (45%)	
**B. Self-Reported NCD Management Approaches among HIV Providers**									
	**Hyperlipidemia**	**Diabetes**	**Hypertension**	**Obesity**	**Osteoporosis**	**Neurocognitive Impairment**	**Sarcopenia**	**Alcohol Use**	**Tobacco Use**
**MD Initial Management**	75 (96%)	74 (95%)	74 (95%)	71 (91%)	61 (78%)	52 (67%)	42 (54%)	41 (53%)	38 (48%)
*primary MD only*	*55 (71%)*	*28 (36%)*	*25 (32%)*	*25 (32%)*	*16 (21%)*	*7 (9%)*	*0 (0%)*	*11 (14%)*	*13 (17%)*
*with specialist referral*	*20 (26%)*	*46 (59%)*	*49 (63%)*	*46 (59%)*	*45 (58%)*	*45 (58%)*	*42 (54%)*	*30 (38%)*	*25 (32%)*
**Specialist Referral Only**	2 (3%)	3 (4%)	3 (4%)	1 (1%)	15 (19%)	24 (31%)	24 (31%)	31 (40%)	34 (44%)
**No Management**	1 (1%)	1 (1%)	1 (1%)	6 (8%)	2 (3%)	2 (3%)	12 (15%)	6 (8%)	6 (8%)

**A. Self-Reported Cancer Screening Approaches.** Screening was categorized as performed by the primary MD if the physician (of any training background) ordered or performed the exam themself. Frequencies reported as n (%). **B. Self-Reported NCD Management Approaches.** Frequencies reported as n (%). *MD Initial management* was defined as any of the following activities: counseling, ordering labs or imaging, reviewing and changing medication regimens as appropriate, pharmacologic interventions, and referral to non-physician ancillary services (ex: nutrition, physical therapy). *Specialist referral only* was defined as any referral to another physician for management (ex: cardiology, psychiatry). Primary HIV providers who both performed initial management activities and referred to another physician specialist for support either for co-management or while their patients waiting for a subspecialist appointment were subcategorized under *MD Initial Management* as above.

### Management practices within the health system context

Health screening practices were put into the health system’s context and separated into screening that was directly recommended by the NHSTIHP HIV Care Guidelines and screening practices which went above and beyond the existing guidelines in Table 3. With the exception of mammography, over 75% of physicians screened patients for conditions recommended by the National Guidelines. By contrast, except for HTN screening, rates were much lower for screening not directly incorporated into the guidelines for HIV care.

**Table 3:** Physician Reported NCD Screening Practices for PLWH According to National Guidelines for HIV Care.

NCD Screening Recommended by National Guidelines	n(%)
Annual glucose screening	64 (82%)
Annual lipid screening	61 (78%)
Annual PAP smear (women)	61 (78%)
Mammography (age 50-69)*	0 (0%)
	
**NCD Screening Beyond the National Guidelines**	**n(%)**
Measure blood pressure each visit	60 (77%)
Calculate BMI at least once a year	39 (50%)
Colon cancer screening (age 40-75)*	0 (0%)
Annual anal PAP smear	3 (4%)
Prostate cancer screening**	17 (22%)
SARC-F score for sarcopenia risk	1 (1%)
FRAX score for fracture risk	4 (5%)
Neurocognitive Screening	19 (24%)

**NCD Screening Practices:** Proportion of physician reported NCD screening practices either performed by themselves OR via a referral. Practices are divided according to whether they are present in the national guidance for HIV care published by the Ministry of Health. *Physicians report that they order these tests in response to symptoms (breast masses, hematochezia etc.), not age-based screening. **Prostate cancer screening was not asked about directly but was noted by physicians during their cancer free response questions.

#### A. NCD screening/management recommended within the NHSTIHP HIV care guidelines.

Diabetes and hyperlipidemia were the most frequently managed by HIV physicians at 95% and 96% respectively, and the most likely to be independently managed without referral (Table 2B). Eighty-two percent of physicians reported ordering annual glucose screening, and 78% ordered annual lipid profiles as recommended by the NHSTIHP HIV care guidelines (Table 3).

Cervical cancer screening was the most widely performed cancer screening (78%) with the assistance of midwives and OB-GYN specialist referrals, but only 10% of physicians would perform a PAP themselves (Table 2A). Breast cancer screening was only ordered or referred for patients who had symptoms such as a breast mass. Although 41% of physicians would do a breast exam or order a mammogram for a breast mass themselves, none of the physicians surveyed reported ordering age-based mammography. Notably, 63% of physicians reported that they would like to order a mammogram, but their patients could not access it (Table 3).

#### B. NCD screening/management beyond the NHSTIHP HIV care guidelines.

*Metabolic diseases:* Hypertension was frequently managed by physicians (95%) either with co-management from a specialist or by themselves (Table 2B). Seventy-seven percent of physicians report measuring blood pressure at every visit (Table 3) and 85% would prescribe an antihypertensive (Fig 3). For diabetes, 73% of physicians would prescribe a hypoglycemic agent, and for hyperlipidemia, 79% would prescribe an ant-lipid agent (Fig 3). Obesity was managed by 91% of physicians either with co-management (63%) or independently (32%) (Table 2B). Fifty percent of physicians reported calculating a BMI for their patients at least annually (Table 3).

*Osteoporosis and Sarcopenia:* While 78% of physicians would attempt some degree of management of osteoporosis, only 21% reported they would do so without additionally placing a specialist referral (Table 2B). While 50% would prescribe vitamin D, only 33% would prescribe an anti-resorptive agent. Only 6% of physicians reported that more than 25% of their patients had age-appropriate bone densitometry. Only 5% of physicians used the FRAX score to calculate risk of osteoporotic-related fractures (Table 3). In their free response answers, physicians noted a lack of availability and affordability of DXA for their patients limited their screening.

Although 54% of physicians reported they would attempt some form of management for sarcopenia, none would do so without consulting a specialist, and 31% percent of physicians would refer their patients to a specialist without attempting any form of management themselves (Table 2B). Ninety-one percent of physicians indicated they did not know the domains to evaluate for sarcopenia and only 1% used the SARC-F score sarcopenia screening (Table 3).

*Non-AIDS defining cancers:* Either by themselves or through referral, 27% of physicians screened their patients for prostate cancer with a prostate specific antigen or direct rectal exam (Table 2A). However, only 4% performed anal PAP smears to screen for anogenital cancer. As with mammography, none of the physicians reported ordering age-based colon cancer screening, although 42% would order or refer for either a colonoscopy or stool occult blood test for symptomatic concerns (Table 3).

*Neurocognitive Impairment:* Even though 83% of physicians had at least one patient with NCI, only 24% of physicians reported they would screen their patients for NCI without being prompted by a symptomatic concern (Table 3). Regarding management approaches, 31% reported that they would refer without attempting to address the NCI and 3% would not address it at all (Table 2B). Of those who attempted to manage NCI, 65% would refer to either psychiatry or neurology, 26% would rule out causes such as depression, co-infection, intoxication, nutritional deficiency, and 24% would order head imaging (Fig 3).

*Tobacco and Alcohol:* Screening practices for alcohol and tobacco were addressed together during this survey. Regarding management approaches, 53% of physicians reported they would do some form of direct management for alcohol use and 48% would do so for tobacco use in their HIV patients. This management mostly took the form of counseling (Fig 3). Forty percent of physicians reported they would refer and not directly address alcohol use and 44% reported the same for tobacco use. For both alcohol use and tobacco use, 8% of physicians reported not addressing the issue at all (Table 2B).

## Discussion

As the global population of people living with HIV ages, we must approach HIV care holistically, addressing both the HIV infection and the non-communicable diseases (NCDs) that are accelerated by aging, chronic inflammation, immune activation, long-term antiviral use, and other biopsychosocial factors such as tobacco and alcohol use [21]. Our results suggest that physicians are generally confident with diseases they are more likely to see in their patients, namely the metabolic diseases (HTN, HLD, DM, obesity). Notable exceptions were alcohol and tobacco use, cervical cancer, and neurocognitive disease, of which the majority physicians encountered at least one case, but reported low confidence, suggesting that these diseases may be a good target for physician training. A minority of physicians encountered at least one patient with osteoporosis, sarcopenia, anal, breast or colon cancer, the lack of encounters with these diagnoses may also be partially due to a lack of screening and diagnosis rather than a true lack of disease. These findings support a need for epidemiologic data on NCDs for PLWH to target high-yield diagnostics and interventions. It is worth highlighting that 63% of physicians would order age-related mammography, but their patients could not afford or access it. Similar issues were noted by the physicians for DXA, which emphasizes a need for accessible screening tools for low resource settings.

Most HIV physicians (>75%) follow the National HIV guidelines with respect to diabetes, hyperlipidemia, and cervical cancer screening, which have readily available screening tests. As resources allow, physicians more consistently screen their patients when such screening is recommended by the national HIV guidelines. Adding additional cancer screening as well as guidance on how to screen for osteoporosis, sarcopenia, and neurocognitive impairment to the national HIV guidelines may increase screening for these NCDs, if systemic barriers to accessing the tests and appropriate training for administering them are simultaneously addressed. Adapting a country and HIV-specific guideline that is as both comprehensive and flexible according to resource context would have a significant impact on standardizing HIV care within Peru and could serve as a model to countries world-wide. Without such guidance, physicians who are caring for HIV patients will struggle with the increasing number of co-morbidities in their aging HIV population.

Integrated NCD and HIV care models have been proposed to reduce care fragmentation and improve the health care experience for multi-morbid HIV patients worldwide, but most studies focus on African countries [22–29]. To our knowledge, there is no literature on successfully implemented integrated NCD-HIV care models in Peru or elsewhere in Latin America. At the time of this study, there are no specific health programs in Peru to comprehensively address the NCD needs of PLWH, but there are general population NCD programs that could be leveraged and adapted to serve PLWH. This study provides important initial groundwork towards understanding the landscape of NCD care for PLWH in Peru. We also identify areas for future study which may be applicable to other countries with similar resource contexts.

This study has some important limitations. We did not have access to patient medical records and were therefore limited to physician report of their practices. Our scope was limited to these particular NCDs and risk factors, which are not the entirety of the NCD burden faced by PLWH; future studies are warranted to address care access and practices for other NCDs such as mental health, renal and pulmonary disease. These results are subject to recall and recency bias, and the limitations of physician self-assessment [30], although a similar physician self-assessment was used to study HIV-Associated Neurocognitive Disorder practices [31]. To minimize recency bias as much as possible, physicians were asked to respond with their “usual” behaviors for their “typical” HIV patient. However, some physicians noted that they did not have patients with less common diseases so their responses to “what would you do,” were theoretical. Finally, physicians who agreed to participate also may have felt more passionately about the importance of non-communicable disease care. As such, this group may be more likely to engage in direct management of NCDs and may feel more confident with non-communicable disease care than other physicians caring for patients with HIV in Peru and management practices may be even more widely varied than depicted here. That said, this study has a valuable national viewpoint, capturing the perspectives of physicians in twenty-three of the twenty-five regions of Peru with a robust survey response rate (47%).

Future research is needed in Peru to review medical records for further practice assessments, conduct a more nuanced assessment of systemic practice barriers, and collect epidemiologic data on patient outcomes to enhance the perspective of this study. Further research is needed on cost-effectiveness and clinical outcomes of integrating NCD and HIV care.

## Conclusions

The majority of physicians in this study are attempting to manage non-communicable diseases among their HIV patients living in Peru even though they may lack confidence and/or knowledge of best management practices. More robust guidelines may enhance NCD management for PLWH, but lack of access to certain screening tests is a barrier for preventing NCDs and must be considered when adapting or expanding existing national HIV care guidelines. Our study highlights the importance of a multifaceted approach to addressing NCD care and the need for further NCD epidemiologic data for PLWH in Peru. Future research and implementation efforts will need to address systemic access barriers to develop locally relevant physician educational resources and create robust NCD management guidelines for PLWH that are adaptable to different resource contexts.

## Supporting information

S1 Data De-identified data file. (XLSX)

S1 Table Inclusion Rationale for Selected Diseases/Risk Factors. (DOCX)

S2 Table Percentage of Physicians who Reported Having at Least One Patient with the Diseases of Interest. (DOCX)

S1 Text Provider Telephone Survey in Spanish. (DOCX)

S2 Text Provider Telephone Survey in English. (DOCX)
