# Supplementary material for: Non-communicable disease care for persons living with HIV in Peru: A national physician cross-sectional study
Source: PLOS Glob Public Health. 2025 Aug 4;5(8):e0004846. doi: 10.1371/journal.pgph.0004846 (PMC12321123; doi:10.1371/journal.pgph.0004846)
Supplement: S1 Table — (DOCX) [file pgph.0004846.s002.docx]

**Supplemental Table 1.**

| **Selected Diseases/Risk Factors** | **Inclusion Rationale** |
| --- | --- |
| ***Osteoporosis*** | PLWH have an increased incidence and earlier onset of osteopenia, osteoporosis, and osteoporosis-related fractures than the general population^1,2^*.* Although similar data for Peru is lacking, a study from Brazil, Columbia, Mexico, and Argentina estimates that by 2026, the annual burden of osteoporosis related fractures in the general population will exceed 1.5 billion USD^3^. |
| ***Sarcopenia*** | In the general population, sarcopenia is a predictor of mortality among the elderly, is associated with physical disability, and increases hospitalization costs. PLWH had a 6.1 greater odds of sarcopenia compared to age-matched controls^4^. |
| ***Hypertension*** | PLWH also have increased rates of hypertension than the general population overall. Among PLWH on ART, who are over the age of 50, an estimated 50% have hypertension^5^. |
| ***Hyperlipidemia:*** | HIV infection, as well as ART, has been associated with dyslipidemia^6,^ which is a risk factor for ischemic stroke and coronary artery disease. In Peru, ischemic stroke and heart disease are the second and third leading cause of death respectively^7^. |
| ***Diabetes*** | Both HIV infection and several classes of ARTs are associated with an increased risk of insulin resistance and diabetes^8^. Diabetes is a modifiable risk factor for cardiovascular disease. |
| ***Non-aids defining cancers:*** | Between the years 2003 and 2016, almost one fifth of deaths were attributed to cancer in Peru^9^. Non-aids defining cancers made up 28% of cancers reported among PLWH in Latin America between 2008-2015^10^. |
| ***Neurocognitive impairment*** | Neurocognitive impairment (impaired memory, language decision making, emotional regulation, motor function or attention) is associated with high morbidity and mortality in PLWH^11^. A recent study in Lima, Peru suggests that upwards of 28.5% of PLWH over the age of 40 years old had NCI and 70% had some deficit in attention or memory^12^. |
| ***Obesity:*** | Although data is lacking for Peru, obesity is prevalent among PLWH in several other Latin American countries^13^. |
| ***Tobacco and Alcohol Use:*** | Both tobacco and alcohol use are major risk factors of multiple non-communicable diseases including cancer, heart disease, osteoporosis, sarcopenia, and diabetes. Tobacco and alcohol use is prevalent in PLWH in Peru^14,15^. |

1. Güerri-Fernandez R, Vestergaard P, Carbonell C, et al. HIV infection is strongly associated with hip fracture risk, independently of age, gender, and comorbidities: a population-based cohort study. *J Bone Miner Res*. 2013;28(6):1259-1263. doi:10.1002/jbmr.1874
2. Prieto-Alhambra D, Güerri-Fernández R, De Vries F, et al. HIV infection and its association with an excess risk of clinical fractures: a nationwide case-control study. *J Acquir Immune Defic Syndr*. 2014;66(1):90-95. doi:10.1097/QAI.0000000000000112
3. Aziziyeh et al., “The Burden of Osteoporosis in Four Latin American Countries: Brazil, Mexico, Colombia, and Argentina.” *Journal of Medical Economics.* 2019;22:7, 638-644, DOI: [10.1080/13696998.2019.1590843](https://doi.org/10.1080/13696998.2019.1590843)
4. Oliveira, V.H.F., Borsari, A.L., Webel, A.R. *et al.* Sarcopenia in people living with the Human Immunodeficiency Virus: a systematic review and meta-analysis. *Eur J Clin Nutr* 74**,**1009–1021 (2020). <https://doi.org/10.1038/s41430-020-0637-0>
5. Fahme SA, Bloomfield GS, Peck R. Hypertension in HIV-Infected Adults: Novel Pathophysiologic Mechanisms. *Hypertension*. 2018;72(1):44-55. doi:10.1161/HYPERTENSIONAHA.118.10893
6. Sarkar S, Brown TT. Lipid Disorders in People with HIV. [Updated 2023 Jan 21]. In: Feingold KR, Anawalt B, Blackman MR, et al., editors. Endotext [Internet]. South Dartmouth (MA): MDText.com, Inc.; 2000-. Available from: https://www.ncbi.nlm.nih.gov/books/NBK567198/
7. Vos, Theo, Stephen S Lim, Cristiana Abbafati, Kaja M Abbas, Mohammad Abbasi, Mitra Abbasifard, Mohsen Abbasi-Kangevari, et al. “Global Burden of 369 Diseases and Injuries in 204 Countries and Territories, 1990–2019: A Systematic Analysis for the Global Burden of Disease Study 2019.” *The Lancet* 396, no. 10258 (October 17, 2020): 1204–22. <https://doi.org/10.1016/S0140-6736(20)30925-9>.
8. Noubissi EC, Katte JC, Sobngwi E. Diabetes and HIV. *Curr Diab Rep*. 2018;18(11):125. Published 2018 Oct 8. doi:10.1007/s11892-018-1076-3
9. Zafra-Tanaka JH, Tenorio-Mucha J, Villarreal-Zegarra D, Carrillo-Larco R, Bernabe-Ortiz A. Cancer-related mortality in Peru: Trends from 2003 to 2016 [published correction appears in PLoS One. 2020 Mar 4;15(3):e0230271]. *PLoS One*. 2020;15(2):e0228867. Published 2020 Feb 6. doi:10.1371/journal.pone.0228867
10. Fink VI, Jenkins CA, Castilho JL, et al. Survival after cancer diagnosis in a cohort of HIV-positive individuals in Latin America. *Infect Agent Cancer*. 2018;13:16. Published 2018 May 8. doi:10.1186/s13027-018-0188-3
11. Banerjee N, McIntosh RC, Ironson G. Impaired Neurocognitive Performance and Mortality in HIV: Assessing the Prognostic Value of the HIV-Dementia Scale. *AIDS Behav*. 2019;23(12):3482-3492. doi:10.1007/s10461-019-02423-w
12. Diaz, Monica M., Marcela Gil Zacarías, Patricia Sotolongo, María F. Sanes, Donald J. Franklin, María J. Marquine, Mariana Cherner, et al. “Characterization of HIV-Associated Neurocognitive Impairment in Middle-Aged and Older Persons With HIV in Lima, Peru.” *Frontiers in Neurology* 12 (2021): 795. <https://doi.org/10.3389/fneur.2021.629257>.
13. Derose KP, Palar K, Farías H, Adams J, Martínez H. Developing Pilot Interventions to Address Food Insecurity and Nutritional Needs of People Living With HIV in Latin America and the Caribbean: An Interinstitutional Approach Using Formative Research. *Food Nutr Bull*. 2018;39(4):549-563. doi:10.1177/0379572118809302
14. Ferro EG, Weikum D, Vagenas P, et al. Alcohol use disorders negatively influence antiretroviral medication adherence among men who have sex with men in Peru. *AIDS Care*. 2015;27(1):93-104. doi:10.1080/09540121.2014.963013
15. Mdege ND, Shah S, Ayo-Yusuf OA, Hakim J, Siddiqi K. Tobacco use among people living with HIV: analysis of data from Demographic and Health Surveys from 28 low-income and middle-income countries. *Lancet Glob Health*. 2017;5(6):e578-e592. doi:10.1016/S2214-109X(17)30170-5
