# Supplementary material for: Non-communicable disease care for persons living with HIV in Peru: A national physician cross-sectional study
Source: PLOS Glob Public Health. 2025 Aug 4;5(8):e0004846. doi: 10.1371/journal.pgph.0004846 (PMC12321123; doi:10.1371/journal.pgph.0004846)
Supplement: S2 Table — (DOCX) [file pgph.0004846.s003.docx]

**Supplemental Table 2**

| **Number of HIV providers who report having any patient with the following condition** | ***n (%)*** |
| --- | --- |
| Hyperlipidemia | 72 (92%) |
| Alcohol use | 72 (92%) |
| Diabetes | 69 (88%) |
| Obesity | 68 (87%) |
| Hypertension | 66 (85%) |
| Neurocognitive Impairment | 65 (83%) |
| Tobacco Use | 62 (79%) |
| Cervical Cancer | 40 (51%) |
| Osteoporosis | 37 (47%) |
| Anal Cancer | 26 (33%) |
| Sarcopenia | 24 (31%) |
| Colon Cancer | 24 (31%) |
| Breast Cancer | 10 (13%) |

**Supplemental Table 2:** Physicians were asked whether they had at least one HIV-positive patient with the diseases of interest: “yes” responses are described above as frequency (precent). These data do not reflect actual disease prevalence within the HIV population of Peru.
