# Supplementary material for: Non-communicable disease care for persons living with HIV in Peru: A national physician cross-sectional study
Source: PLOS Glob Public Health. 2025 Aug 4;5(8):e0004846. doi: 10.1371/journal.pgph.0004846 (PMC12321123; doi:10.1371/journal.pgph.0004846)
Supplement: S1 Text — (DOCX) [file pgph.0004846.s004.docx]

**S1 Text:** Peruvian HIV Provider Telephone Survey, Spanish

Q1 ID en la Encuesta:

Q2 (*Confirmar)*Ubicación de centro de salud:

**Parte I.** Comenzaremos con información demográfica básica para asegurarnos de que tenemos opiniones de diversos orígenes.

Q3 Cual es tu edad?

Q4 Cuál es tu género?

Q5 Número de años en la práctica médica?

Q6 Número de años como un medico general?

Q7 Número de años como especialista?

Q8 ¿Cuál fue su programa de formación?

Q9 ¿Cuántos pacientes con VIH ha visto el mes pasado?

Q10 ¿Qué porcentaje de pacientes con VIH que usted atiende son mayores de 40 años?

Q11 ¿Alguno de sus pacientes con VIH padece de alguna de las siguientes condiciones? *(Marque todas las que corresponda):*

| Osteoporosis | - Si | - No | - No lo sé |
| --- | --- | --- | --- |
| Sarcopenia | - Si | - No | - No lo sé |
| Hipertensión | - Si | - No | - No lo sé |
| Diabetes | - Si | - No | - No lo sé |
| Obesidad | - Si | - No | - No lo sé |
| Hiperlipidemia | - Si | - No | - No lo sé |
| Alteración neurocognitiva | - Si | - No | - No lo sé |
| Cáncer de mama | - Si | - No | - No lo sé |
| Cáncer de colon | - Si | - No | - No lo sé |
| Cáncer anal | - Si | - No | - No lo sé |
| Cáncer cervical | - Si | - No | - No lo sé |
| Consumo de Tabaco | - Si | - No | - No lo sé |
| Consumo de Alcohol | - Si | - No | - No lo sé |
| Otros (*por favor liste cuales)* |  | | |

**Parte II.** Q12. Nivel de seguro respecto a enfermedades no transmisibles en personas con VIH “Las siguientes preguntas le pedirán que evalúe qué tan cómodo se siente. En las siguientes preguntas le voy a pedir que usando una escala de 1 al 4, en donde 1 es no seguo 2 poco seguro 3 seguro y 4 muy seguro, me diga que tan seguro se siente para manejar las siguientes enfermedades no transmisibles en personas con VIH."

| Enfermedad No Transmisible | Evaluación de Nivel de comodidad  *Evalúe “tamizaje”, “diagnóstico” y “prevención” con_*  *1 = no seguro*  *2 = poco seguro*  *3 = seguro*  *4= muy seguro* | | |
| --- | --- | --- | --- |
| Osteoporosis | Prevención= **_______** | Diagnóstico= **_______** | Tratamiento= ____________ |
| Sarcopenia | Prevención= **_______** | Diagnóstico= **_______** | Tratamiento= ____________ |
| Obesidad | Prevención= **_______** | Diagnóstico= **_______** | Tratamiento= ____________ |
| Diabetes | Prevención= **_______** | Diagnóstico= **_______** | Tratamiento= ____________ |
| Hipertensión | Prevención= **_______** | Diagnóstico= **_______** | Tratamiento= ____________ |
| Hiperlipidemia | Prevención= **_______** | Diagnóstico= **_______** | Tratamiento= ____________ |
| Cáncer cervical | Prevención= **_______** | Diagnóstico= **_______** | Tratamiento= ____________ |
| Alteración neurocognitiva | Prevención= **_______** | Diagnóstico= **_______** | Tratamiento= ____________ |
| Consumo de Alcohol | Prevención= **_______** | Diagnóstico= **_______** | Tratamiento= ____________ |
| Consumo de Tabaco | Prevención= **_______** | Diagnóstico= **_______** | Tratamiento= ____________ |

**Parte III:** Preguntas de Tendencias de Consulta Clínica:
*“Las siguientes preguntas se relacionan a su experiencia en la práctica clínica con pacientes con VIH, no hay respuestas malas, si no lo hace o desconoce dígalo."*

Q13. Osteoporosis: ¿Qué porcentaje de sus pacientes varones mayores de 50 años con VIH y mujeres postmenopáusicas con VIH han sido evaluados con una densitometría ósea?

- No tengo ningún paciente con estos rangos de edades
- No manejo este problema
- ≤25%
- 26- 50%
- 51-75%
- ≥ 76%

Q14. Osteoporosis: Si uno de sus pacientes con VIH tuviera osteoporosis, ¿qué acciones tomaría? *Respuesta libre, no se leyeron las opciones de respuesta a los participantes.*

- Normalmente no manejo este problema
- Examino los niveles de vitamina D y Calcio
- Aconsejo ejercicios con peso y disminución de consumo de alcohol y tabaco
- Recomiendo/prescribo vitamina D y Calcio
- Me siento cómodo prescribiendo bifosfonatos si lo amerita
- Refiero a un especialista para el manejo de osteoporosis
- Refiero a un especialista solo si hay una causa secundaria de osteoporosis o si hay una fractura asociada
- No sé
- Revise su TAR y cámbielo si es necesario
- Otros ________________________________________________

Q15 Osteoporosis ¿Ha utilizado el score de FRAX en sus pacientes con VIH para tamizar el riesgo de fractura asociada a osteoporosis?

- Si
- No
- No tengo pacientes para los que este score sea relevante
- No lo conozco

Q16. Sarcopenia ¿Conoce cuáles son los dominios para evaluar Sarcopenia? ¿Podría, podría listarlos?

*Respuesta libre, no se leyeron las opciones de respuesta a los participantes.*

- Fuerza – habilidad para levantar 4.5 kilogramos
- Habilidad para caminar por una habitación
- Habilidad para levantarse de una silla
- Habilidad para subir 10 escalones
- Historia de caídas
- Todas las anteriores
- No conozco

Q17. Sarcopenia Si usted tuviera un paciente con VIH y sarcopenia, ¿qué medidas tomaría? *Respuesta libre, no se leyeron las opciones de respuesta a los participantes:*

- Normalmente no manejo este problema
- Examino los niveles de Vitamina D
- Recomiendo/prescribo Vitamina D
- Recomiendo ejercicio físico
- Evalúo la ingesta proteica y sugiero aumentar su consumo
- Recomiendo suplementos proteicos nutricionales
- Refiero a un especialista para manejo de sarcopenia
- Refiero a un especialista solo si la sarcopenia persiste a pesar del tratamiento dado o si el paciente presenta sarcopenia severa
- No sé
- Otros ________________________________________________

Q18. Sarcopenia ¿Utiliza el score SARC-F para tamizar sarcopenia en sus pacientes con VIH?

- Si
- No
- No tengo pacientes para los que este score sea relevante
- No lo conozco

Q20. Hipertensión Si uno de sus pacientes con VIH viene a consulta con al menos dos mediciones de presión arterial >140/90 , ¿Como lo manejaria? *Respuesta libre, no se leyeron las opciones de respuesta a los participantes:*

- Normalmente no manejo este problema
- Aconsejo a los pacientes sobre los riesgos de una presión arterial elevada
- Aconsejo cambios de estilos de vida y dieta para disminuir la presión arterial
- Prescribo medicación para disminuir la presión arterial
- Refiero al paciente a un especialista para manejo antihipertensivo
- Refiero a un especialista solo si no logro manejar la presión arterial con medicamentos
- No sé
- Ordeno otros examenes para evaluar otras comorbilidades
- Otros ________________________________________________

Q21. Diabetes: ¿A qué porcentaje de sus pacientes con VIH en TARGA tienen un examen de glucosa en ayunas al menos una vez al año como monitoreo de su tratamiento?

- No manejo este problema
- ≤25%
- 26- 50%
- 51-75%
- ≥ 76%

Q22. Diabetes Para pacientes con VIH con una glucosa en ayunas ≥ 126mg/dl, ¿qué acciones tomaría? *Respuesta libre, no se leyeron las opciones de respuesta a los participantes:*

- Normalmente no manejo este problema
- Aconsejo a los pacientes sobre los riesgos de diabetes mellitus
- Aconsejo cambios de estilos de vida y dieta para disminuir la glucosa en sangre
- Prescribo medicación para disminuir la glucosa en sangre
- Refiero al paciente a un especialista para manejo de diabetes mellitus
- Refiero a un especialista solo si no logro controlar la glucosa con medicamentos.
- Refiero a un nutricionista
- No sé
- Otros ________________________________________________

Q23. Hiperlipidemia: ¿Qué porcentaje de sus pacientes con VIH en TARGA tienen un perfil lipídico anual como monitoreo de su tratamiento?

- No manejo este problema
- ≤25%
- 26- 50%
- 51-75%
- ≥ 76%

Q24. Hiperlipidemia: Si tiene un paciente con VIH y un LDL ≥ 190, ¿Qué acciones tomaría? *Respuesta libre, no se leyeron las opciones de respuesta a los participantes:*

- Normalmente no manejo este problema
- Aconsejo al paciente sobre los riesgos de la hiperlipidemia
- Aconsejo sobre cambios de estilos de vida para disminuir los niveles de LDL
- Prescribo medicación para disminuir los niveles de LDL
- Refiero a un especialista para manejo de hiperlipidemia
- Refiero a un especialista solo si no puedo controlar la hiperlipidemia con medicación
- Refiero a un nutricionista
- No sé
- Otros ________________________________________________
- Revise su TAR y cámbielo si es necesario

Q25. Obesidad ¿Con qué frecuencia calcula el Índice de Masa Corporal (IMC) de sus pacientes?

- En cada vista
- Solo en la primera visita
- 1 vez al año o más frecuentemente
- Cada 2 años o menos frecuente
- Solo cuando el paciente lo solicita
- Nunca
- Solo por un signo / historia de aumento / pérdida de peso

Q26. Obesidad Si tiene un paciente con VIH y Índice de Masa Corporal >25, ¿Qué acciones tomaría?  *Respuesta libre, no se leyeron las opciones de respuesta a los participantes:*

- Normalmente no manejo este problema
- No tengo pacientes con IMC >25
- Discutir sobre estrategias de disminución de peso
- Discutir sobre actividad física del paciente
- Discutir sobre la nutrición del paciente
- Prescribo medicamentos o suplementos para bajar de peso
- Crear un plan centrado en el paciente para la disminución de peso
- Refiero a un nutricionista
- No sé
- Otros ________________________________________________

Q27. Cáncer: Sobre el tamizaje de cáncer en las mujeres con VIH, ¿Qué acciones tomaría?  *Respuesta libre, no se leyeron las opciones de respuesta a los participantes:*

- Normalmente no manejo este problema
- Mis pacientes son muy jóvenes
- Refiero a un especialista para el tamizaje
- Ordeno una mamografía cada 2 años para pacientes de 50-74 años
- Ordenaría una mamografía, pero mis pacientes no pueden acceder a este examen (por ejemplo: viven muy lejos, es muy caro, la lista de espera es muy larga)
- Realizo un examen físico mamario
- Ordeno una colonoscopia cada 10 años para pacientes de 45-75 años
- Ordenaría una colonoscopia, pero mis pacientes no pueden acceder a este examen (por ejemplo: viven muy lejos, es muy caro, la lista de espera es muy larga)
- Ordeno un examen de sangre oculta en heces anualmente para pacientes con edades de 45-75 años
- Ordeno un examen inmunohistoquímico de sangre oculta en heces anualmente para pacientes con edades de 45-75 años
- Realizo un examen PCR de VPH para mujeres de edad ≥ 30 años y si es negativo, lo repito cada 3 años
- Ofrezco la vacuna del VPH si no ha sido vacunado previamente
- Aconsejo a no consumir tabaco
- Aconsejo a no consumir alcohol
- No sé
- Otros ________________________________________________

Q28. Cáncer: Sobre el tamizaje de cáncer en los hombres con VIH, ¿Qué acciones tomaría?

*Respuesta libre, no se leyeron las opciones de respuesta a los participantes:*

- Normalmente no manejo este problema
- Mis pacientes son muy jóvenes
- Refiero a un especialista para el tamizaje
- Ordeno una colonoscopia cada 10 años para pacientes de 45-75 años
- Ordenaría una colonoscopia, pero mis pacientes no pueden acceder a este examen (por ejemplo: viven muy lejos, es muy caro, la lista de espera es muy larga)
- Ordeno un examen de sangre oculta en heces anualmente para pacientes con edades de 45-75 años
- Ordeno un examen inmunohistoquímico de sangre oculta en heces anualmente para pacientes con edades de 45-75 años
- Realizo un Papanicolau anal para pacientes con historia de sexo anal o con resultado anormal de Papanicolau cervical
- Ofrezco la vacuna del VPH si no ha sido vacunado previamente
- Aconsejo a no consumir tabaco
- Aconsejo a no consumir alcohol
- No sé
- Otros ________________________________________________

Q29. Alteración Neurocogn ¿Con qué frecuencia pregunta a sus pacientes con VIH si es que tienen algún problema de alteración cognitiva (ex: memoria, lenguaje, toma de decisiones, regulación emocional, función motora, atención)?

- Normalmente no manejo este problema
- Solo en la visita inicial
- Cada 6 meses o más frecuentemente
- Cada año y menos frecuentemente
- Solo cuando es necesario como parte de la evaluación de otros síntomas

Q30. Alteración Neurocogn Si tuviera a un paciente con VIH con evidencia de alteración cognitiva, ¿Qué acciones tomaría?

*Respuesta libre, no se leyeron las opciones de respuesta a los participantes:*

- Normalmente no manejo este problema
- Evaluar si un cambio de tratamiento antirretroviral es necesario
- Tratar de no usar medicamentos sedantes
- Realizar un test cognitivo breve como el MOCA, MMSE o la escala de demencia en VIH
- Preguntar por miembros de familia o algún otro asistente que pueda ayudar con sus medicamentos
- Referir al neuropsiquiatra
- Referir a un neurólogo
- Referir a un geriatra
- No sé
- Otros ________________________________________________

Q31. Estilos de Vida Si tuvo un paciente con VIH que consume tabaco o consume alcohol, ¿qué acciones tomaría?

*Respuesta libre, no se leyeron las opciones de respuesta a los participantes:*

- Normalmente no manejo este problema
- No tengo pacientes que consuman alcohol o tabaco
- Mis pacientes no hablan de estos temas conmigo
- Tengo pacientes que consumen alcohol o tabaco, pero no sé como ayudarlos a que se detengan
- Los aconsejo a que no consuman tabaco
- Ordeno un “reemplazo de nicotina”
- Refiero al paciente a un especialista o a algún grupo de soporte por consumo de tabaco
- Aconsejo sobre los beneficios de limitar el consumo de alcohol
- Aconsejo sobre los beneficios de dejar de consumir alcohol
- Refiero a un especialista o a algún grupo de soporte por consumo de alcohol
- Mis pacientes que consumen alcohol o tabaco no tienen acceso a ningún recurso que los ayude a detener su consumo
- No sé
- Otros ________________________________________________

Cierre ¡GRACIAS POR SU VALIOSA PARTICIPACIÓN! FIN.
