# Supplementary material for: Non-communicable disease care for persons living with HIV in Peru: A national physician cross-sectional study
Source: PLOS Glob Public Health. 2025 Aug 4;5(8):e0004846. doi: 10.1371/journal.pgph.0004846 (PMC12321123; doi:10.1371/journal.pgph.0004846)
Supplement: S2 Text — (DOCX) [file pgph.0004846.s005.docx]

**S2 Text:** Peruvian HIV Provider Telephone Survey, English

Q1 Survey ID:

Q2 (*Confirm)* Health Center Location:

**Part I.** *We'll start with basic demographic information to make sure we have opinions from diverse backgrounds:*

Q3: What is your age?

Q4: What is your gender?

Q5: How many years have you been in medical practice?

Q6: (*if applicable):* How many years have you worked as a general practitioner?

Q7: (*if applicable):* How many years have you worked as a specialist?

Q8: What was your training program?

Q9: How many HIV patients have you seen in the past month?

Q10: What percentage of HIV patients that you care for are over the age of 40?

Q11 Do any of your HIV patients have any of the following conditions? *(Check all that apply):*

| Osteoporosis | - Yes | - No | - I don’t know |
| --- | --- | --- | --- |
| Sarcopenia | - Yes | - No | - I don’t know |
| Hypertension | - Yes | - No | - I don’t know |
| Diabetes | - Yes | - No | - I don’t know |
| Obesity | - Yes | - No | - I don’t know |
| Hyperlipidemia | - Yes | - No | - I don’t know |
| Neurocognitive Disorder | - Yes | - No | - I don’t know |
| Breast Cancer | - Yes | - No | - I don’t know |
| Colon Cancer | - Yes | - No | - I don’t know |
| Anal Cancer | - Yes | - No | - I don’t know |
| Cervical Cancer | - Yes | - No | - I don’t know |
| Tobacco Use | - Yes | - No | - I don’t know |
| Alcohol Use | - Yes | - No | - I don’t know |
| Other *(please list)* |  | | |

**Part II.** Q12. Provider confidence level with respect to non-communicable diseases in PLWH. “The following questions ask you to evaluate your comfort level using a scale from one to four where 1 is “no confidence”, 2 is “little confidence”, 3 is “confident”, and 4 is “very confident.” Tell us how confident you feel managing the following non-communicable diseases in PLWH:

| Non-communicable Disease/Risk Factor | Evaluate level of confidence for screening, diagnosis, and prevenion:  *1 = no confidence*  *2 = little confidence*  *3 = confident*  *4= very confident* | | |
| --- | --- | --- | --- |
| Osteoporosis | Prevention= **_______** | Diagnosis= **_______** | Treatment= ____________ |
| Sarcopenia | Prevention= **_______** | Diagnosis= **_______** | Treatment= ____________ |
| Obesity | Prevention= **_______** | Diagnosis= **_______** | Treatment= ____________ |
| Diabetes | Prevention= **_______** | Diagnosis= **_______** | Treatment= ____________ |
| Hypertension | Prevention= **_______** | Diagnosis= **_______** | Treatment= ____________ |
| Hyperlipidemia | Prevention= **_______** | Diagnosis= **_______** | Treatment= ____________ |
| Cervical Cancer | Prevention= **_______** | Diagnosis= **_______** | Treatment= ____________ |
| Neurocognitive Disorder | Prevention= **_______** | Diagnosis= **_______** | Treatment= ____________ |
| Alcohol Use | Prevention= **_______** | Diagnosis= **_______** | Treatment= ____________ |
| Tobacco Use | Prevention= **_______** | Diagnosis= **_______** | Treatment= ____________ |

**Part III:** Clinical Practice Management Questions:
*"The following questions relate to your experience in clinical practice with HIV patients. There are no bad answers; if you don't do or know something, just say so."*

Q13. Osteoporosis: What percentage of your male patients over the age of 50 with HIV and postmenopausal women with HIV have been evaluated with bone densitometry?

- I don't have any patients with these age ranges
- I don't manage this
- ≤25%
- 26- 50%
- 51-75%
- ≥ 76%

Q14. Osteoporosis: If one of your HIV patients had osteoporosis, what actions would you take? *Free response, the answer options were not read to the participants.*

- I don't normally handle this problem
- I test vitamin D and calcium levels
- I advise weight-bearing exercise and a reduction in alcohol and tobacco consumption
- I recommend/prescribe Vitamin D and Calcium
- I'm comfortable prescribing bisphosphonates if warranted
- I refer to a specialist for the management of osteoporosis
- I refer to a specialist only if there is a secondary cause of osteoporosis or an associated fracture
- I do not know
- Check their ART regimen and change it if needed
- Other________________________________________________

Q15 Osteoporosis: Have you used the FRAX score in your HIV patients to screen for osteoporosis-associated fracture risk?

- Yes
- No
- I don't have patients for whom this score is relevant
- I don't know it

Q16. Sarcopenia: Do you know which domains to evaluate for sarcopenia? Please list them:

*Free response, the answer options were not read to the participants.*

- Strength – ability to lift 4.5 kilograms
- Ability to walk around a room
- Ability to get up from a chair
- Ability to climb 10 steps
- History of Falls
- All of the above
- I don't know

Q17. Sarcopenia: If you had a patient with HIV and sarcopenia, what steps would you take? *Free response, the response options were not read to the participants:*

- I don't normally handle this problem
- I test Vitamin D levels
- I recommend/prescribe Vitamin D
- I recommend physical exercise
- I evaluate protein intake and suggest increasing your intake
- I recommend nutritional protein supplements
- I refer to a specialist for sarcopenia management
- I refer to a specialist only if sarcopenia persists despite treatment or if the patient has severe sarcopenia
- I do not know
- Other________________________________________________

Q18. Sarcopenia: Do you use the SARC-F score to screen for sarcopenia in your HIV patients?

- Yes
- No
- I don't have patients for whom this score is relevant
- I don't know it

Q19. Hypertension: How often do you check the blood pressure of your HIV patients?

- At every encounter
- Only at the first encounter
- Once a year or more frequently
- Every 3-5 years or less
- Only when necessary for some related symptom/sign/history
- Never

Q20. If one of your HIV patients comes to the clinic with at least two blood pressure measurements >140/90, how would you handle it? *Free response, the response options were not read to the participants:*

- I don't normally handle this problem
- I advise patients about the risks of high blood pressure
- I advise lifestyle and diet changes to lower blood pressure
- I prescribe medication to lower blood pressure
- I refer the patient to a specialist for antihypertensive management
- I refer to a specialist only if I can't manage my blood pressure with medication
- I do not know
- I order other tests to evaluate other comorbidities
- Other________________________________________________

Q21. Diabetes: What percentage of your HIV patients in HAART have a fasting glucose test at least once a year as monitoring for their treatment?

- I don't handle this problem
- ≤25%
- 26- 50%
- 51-75%
- ≥ 76%

Q22. Diabetes: For HIV patients with a fasting glucose ≥ 126mg/dl, what actions would you take? *Free response, the response options were not read to the participants:*

- I don't normally handle this problem
- I advise patients about the risks of diabetes mellitus
- I advise lifestyle and diet changes to lower blood glucose
- I prescribe medication to lower blood glucose
- I refer the patient to a specialist for diabetes mellitus management
- I refer to a specialist only if I can't control my glucose with medication.
- I refer to a nutritionist
- I do not know
- Other________________________________________________

Q23. Hyperlipidemia: What percentage of your HIV patients in HAART have an annual lipid profile as a monitoring of their treatment?

- I don't handle this problem
- ≤25%
- 26- 50%
- 51-75%
- ≥ 76%

Q24. Hyperlipidemia: If you have a patient with HIV and an LDL ≥ 190, what actions would you take? *Free response, the response options were not read to the participants:*

- I don't normally handle this problem
- I advise the patient about the risks of hyperlipidemia
- I advise on lifestyle changes to lower LDL levels
- I prescribe medication to lower LDL levels
- I check their ART regimen and change it if needed
- I refer to a specialist for hyperlipidemia management
- I refer to a specialist only if I can't control hyperlipidemia with medication
- I refer to a nutritionist
- I do not know
- Other________________________________________________

Q25. Obesity: How often do you calculate your patients' Body Mass Index (BMI)?

- At each encounter
- Only on the first encounter
- Once a year or more frequently
- Every 2 years or less often
- Only when requested by the patient
- Just for a sign/history of weight gain/loss
- Never

Q26. Obesity: If you have a patient with HIV and Body Mass Index >25, what actions would you take?  *Free response, the response options were not read to the participants:*

- I don't normally handle this problem
- I don't have patients with BMI >25
- I discuss weight loss strategies
- I discuss patient’s physical activity
- I discuss the patient’s nutrition
- I prescribe medications or supplements for weight loss
- I create a patient-centered plan for weight loss
- I refer to a nutritionist
- I do not know
- Other________________________________________________

Q27. Cancer: What actions would you take about cancer screening in female patients with HIV?  *Free response, the response options were not read to the participants:*

- I don't normally handle this problem
- My patients are too young
- I refer to a specialist for screening
- I order a mammogram every 2 years for patients ages 50-74
- I would order a mammogram, but my patients can't access this test (e.g. they live too far away, it's very expensive, the waiting list is too long)
- I perform a breast physical exam
- I order a colonoscopy every 10 years for patients ages 45-75
- I would order a colonoscopy, but my patients can't access this exam (for example: they live very far away, it's very expensive, the waiting list is very long)
- I order a fecal occult blood test annually for patients ages 45-75
- I order an immunohistochemical examination of fecal occult blood annually for patients aged 45-75 years
- I perform a cervical PAP smear
- I offer the HPV vaccine if they have not been vaccinated previously
- I advise against tobacco use
- I advise against alcohol use
- I do not know
- Other________________________________________________

Q28. Cancer: On cancer screening in male patients with HIV, what actions would you take?

*Free response, the response options were not read to the participants:*

- I don't normally handle this problem
- My patients are too young
- I refer to a specialist for screening
- I order a colonoscopy every 10 years for patients ages 45-75
- I would order a colonoscopy, but my patients can't access this exam (for example: they live very far away, it's very expensive, the waiting list is very long)
- I order a fecal occult blood test annually for patients ages 45-75
- I order an immunohistochemical examination of fecal occult blood annually for patients aged 45-75 years
- Performed an anal Pap smear
- I offer the HPV vaccine if they have not been vaccinated previously
- I advise against tobacco use
- I advise against alcohol use
- I do not know
- Other________________________________________________

Q29. How often do you ask your HIV patients if they have any problems with cognitive impairment (e.g., memory, language, decision-making, emotional regulation, motor function, attention)?

- I don't normally handle this problem
- Only at the initial encounter
- Every 6 months or more frequently
- Every year and less frequently
- Only when needed as part of the evaluation of other symptoms

Q30. If you had an HIV patient with evidence of cognitive impairment, what actions would you take?

*Free response, the response options were not read to the participants:*

- I don't normally handle this problem
- Assess whether a change in antiretroviral treatment is necessary
- Try not to use sedative medications
- Take a brief cognitive test such as the MOCA, MMSE, or the HIV Dementia Scale
- Ask for family members or other assistants who can help with their medications
- Refer to a neuropsychiatrist
- Refer to a neurologist
- Refer to a geriatrician
- I do not know
- Other________________________________________________

Q31. Lifestyle: If you had a patient with HIV who uses tobacco or alcohol, what actions would you take?

*Free response, the response options were not read to the participants:*

- I don't normally handle this problem
- I don't have patients who use alcohol or tobacco
- My patients don't talk about these issues with me
- I have patients who use alcohol or tobacco, but I don't know how to help them stop
- I advise them not to use tobacco
- I order a "nicotine replacement"
- I refer the patient to a specialist or a tobacco support group
- I advise on the benefits of limiting alcohol consumption
- I advise on the benefits of quitting alcohol consumption
- I refer to a specialist or an alcohol support group
- My patients who use alcohol or tobacco don't have access to any resources to help them stop their use
- I do not know
- Other________________________________________________

THANK YOU FOR YOUR PARTICIPATION!
